# Food neophobia and its relation with olfaction

**DOI:** 10.3389/fpsyg.2014.00127

**Published:** 2014-02-17

**Authors:** M. Luisa Demattè, Isabella Endrizzi, Flavia Gasperi

**Affiliations:** Department of Food Quality and Nutrition, Research and Innovation Centre, Fondazione Edmund Mach, San Michele all’AdigeItaly

**Keywords:** food neophobia, olfaction, food exposure, odor identification, explorative behavior

## Abstract

Food neophobia, that is the reluctance to try novel foods, is an attitude that dramatically affects human feeding behavior in many different aspects among which food preferences and food choices appear to be the most thoroughly considered. This attitude has an important evolutionary meaning since it protects the individual from ingesting potentially dangerous substances. On the other hand, it fosters an avoidance behavior that can extend even toward useful food elements. A strong link exists between food neophobia and both the variety in one person’s diet and previous exposures to different foods. In this review, the more recent findings about food neophobia will be concisely described. Given the suggested connection between the exposure to different foods and food neophobia, this review will focus on the relation between this attitude and human chemosensory abilities. Olfaction, in particular, is a sensory modality that has a central role in flavor perception and in food preference acquisition. Therefore, the latest evidences about its relation with food neophobia will be discussed along with the applied and cognitive implications.

## INTRODUCTION

Human feeding behavior is guided by a number of different factors relating to the properties of both food product and individual. The intrinsic sensory properties of food are fundamental in modulating the experience the individual has while approaching and consuming the product (Desor et al., 1975). On the other hand, the physiological state of the organism (e.g., hunger; Rolls, 2012) promotes or inhibits food research and consumption (Small et al., 2001; Albrecht et al., 2009; Fernandez et al., 2013). Another extremely important aspect is represented by the cognitive and motivational factors of the individual (Assanand et al., 1998), among which the tendency to avoid foods never encountered before (known as food neophobia; Pliner and Hobden, 1992) is receiving increased attention. The rationale behind this is the existence of a strong connection between new food avoidance with the successive development of unhealthy eating habits (e.g., assuming too much fats or sugars), that can have serious negative consequences on diet balancing or on body weight (e.g., obesity; Capiola and Raudenbush, 2012). Therefore, the purpose of this review is to provide an up-to-date overview of the findings in food neophobia investigation and in the study of its relationship with chemosensory perception, focusing on odor perception.

## ATTITUDES TOWARD FOOD: THE CASE OF FOOD NEOPHOBIA

Among the psychological factors modulating an individual’s relationship with food, the systematic reluctance to try novel or unknown foods (i.e., food neophobia; Pliner and Hobden, 1992) appears to play a critical role in the development of possible eating disorders (see Benton, 2004). From an adaptive point of view, food neophobia protects an organism (animal or human being) from ingesting potentially dangerous foods. This mechanism has a cost, though, represented by the risk of avoiding even highly nutritious foods. The balance an organism should find between these two opposite pressures is known as the “omnivore’s dilemma” (Rozin and Vollmecke, 1986). Since the late 1960s, a large body of research has been produced on this behavior in animals (see e.g., Rozin, 1968; Roberts and Cheney, 1974; Mitchell et al., 1975), whilst food neophobia in humans has only been extensively investigated in the last two decades (for an earlier review, see Frank and Raudenbush, 1998).

In order to try and quantify this in human beings, over the years a number questionnaires have been developed such as the “Food Attitude Survey” (FAS; Frank and van der Klaauw, 1994; see also Frank and Raudenbush, 1998; Raudenbush et al., 1998), but it is with the publication of the “Food Neophobia Scale” (FNS; Pliner and Hobden, 1992) that a systematic way of studying food neophobia initiated. This scale has been successfully used to predict people’s attitude toward new foods and the expected liking of food products, and has been adapted for children administration (“Children Food Neophobia Scale”, CFNS; Pliner, 1994). It has also been translated into different languages and cultures (e.g., for Italian, see Demattè et al., 2013; for Spanish, see Fernández-Ruiz et al., 2013; for Chilean, see Schnettler et al., 2013; for Finnish, see Tuorila et al., 2001; for Japanese, see Yamada et al., 2012). Recently, the FNS has also been adapted to the fruit and vegetable domain (“Fruit and Vegetable Neophobia Instrument”, FVNI; Hollar et al., 2013).

The strength of the FNS lies in the speed at which the questionnaire can be administered, by means of both paper and pencil and computerized tests, and in its repeatedly proven internal consistency (Pliner and Hobden, 1992; Tuorila et al., 1994; Raudenbush et al., 1998). A disadvantage of the scale is that, despite the increasing number of studies performed, a common reliable methodology to use to categorize people as a function of the degree of neophobia is still not available (Meiselman et al., 2010). The FNS can be used to determine neophobia classes by using one standard deviation from the group mean as the splitting criterion (Pliner and Hobden, 1992; Falciglia et al., 2000; Tuorila et al., 2001), by median split (Mustonen et al., 2012; Raudenbush and Capiola, 2012; Yamada et al., 2012), or else by tertiles split (Raudenbush et al., 2003; Tuorila and Mustonen, 2010; Capiola and Raudenbush, 2012; Fernández-Ruiz et al., 2013). Additional new approaches have also been tested recently, for example the segmentation based on Principal Component Analysis (PCA; Demattè et al., 2013; Fernández-Ruiz et al., 2013).

## FOOD NEOPHOBIA AND INDIVIDUAL FACTORS

A large number of individual factors have shown to be connected to the degree of food neophobia. Knaapila et al. (2011) reported that (especially in women) this attitude appears to be strongly genetically determined. The results of the studies conducted so far on gender differences are still quite inconclusive: Some authors have found that women are more neophobic than men (Frank and van der Klaauw, 1994), some authors described instead the contrary (Tuorila et al., 2001), whilst some others failed to find any differences at all (Flight et al., 2003; Nordin et al., 2004; Meiselman et al., 2010; Demattè et al., 2013). A clearer link has instead been described between food neophobia and age. Avoidance behavior of unfamiliar foods would appear and reach its maximum between 2 and 6 years of age (Raudenbush et al., 1998; Blissett and Fogel, 2013), starting from toddlers’ developmental phase of increased physical and motor skills when they gain potential access to a larger number of (possibly dangerous) food substances (Benton, 2004). From late childhood, the levels of neophobia gradually decrease until adulthood, when this tendency would reach its minimum level (Fernández-Ruiz et al., 2013; Schnettler et al., 2013). With aging, food neophobia levels slowly start to rise again (Tuorila et al., 2001), protecting the weaker elderly organism from potential poisoning (Dovey et al., 2008). From a more psychological perspective, studies have highlighted that neophobic people would be less prone to look for strong emotions and adventures (Otis, 1984), more anxious (Dovey et al., 2008), and less open (Knaapila et al., 2011).

## FEEDING BEHAVIOR AND THE ROLE OF OLFACTION

Olfaction plays a crucial role in human life. It has special connections to those areas in the brain involved in the processing and encoding of emotions and memories (Royet et al., 2003), thus it is extremely relevant in human social interaction (see e.g.; Herz and Inzlicht, 2002; Schaal et al., 2004; Demattè et al., 2007). Its importance extends also to the production of adaptive behaviors in response to the environmental stimulations. Olfaction works with the double function of alerting the organism for potentially dangerous elements present in the environment and recognizing foods useful for survival (Prescott, 1999). It is extremely influential on feeding as it represents a basic piece of flavor perception (Small et al., 1997; Prescott, 2012). As a matter of fact, flavor perception (that is the multisensory experience par excellence; Small, 2012), can be disrupted by a simple cold. While perception of the different tastes remains unaltered allowing sweetness to emerge from a candy, the information about the peach flavor of that candy gets lost in the air flow that cannot reach the olfactory epithelium. Therefore, odors appear to be crucial when it comes to the sensory evaluation of a food (Yeomans, 2006).

The investigation of chemosensory functions in eating behavior has mainly taken into account the possible differences in odor functions of patients suffering from eating disorders (e.g., anorexia) and control participants. The results described so far are not always consistent as different groups of people and different methods have been used. For instance, a study reported that people suffering from anorexia nervosa (Roessner et al., 2005) had higher olfactory thresholds and poorer discrimination abilities (but preserved odor identification performance; see also Kopala et al., 1995) than controls. On the contrary in a more recent work, anorectic patients showed to have impaired odor identification abilities (Rapps et al., 2010) with preserved olfactory thresholds. Additionally, there exist other studies targeting obese participants while focusing on taste perception rather than on olfaction. Some of the basic tastes (e.g., salty) seem to have significantly higher thresholds in obese than control participants (Overberg et al., 2012), even though others failed to show any variations (for a review, see Donaldson et al., 2009). However, for odors, there is still no evidence of the existence of reliable differences in perception in obese patients.

A different area of investigation in the field of feeding considers instead the existence of differences in the hedonic evaluation of target stimuli. The evidences indicate that people suffering from anorexia consistently perceive both odors and tastes as less pleasant than control participants (Simon et al., 1993; Jiang et al., 2010). Obese people instead do not seem to show any consistent variations in the hedonic evaluation of chemosensory stimuli (Thompson et al., 1977; Malcolm et al., 1980; though see Drewnowski et al., 1985). A significant difference seems to emerge when looking at the rewarding value of such stimuli during real food consumption. As a matter of fact in a fMRI study, a group of obese girls showed, during both food consumption and anticipation of intake, more neuronal activity than controls in those areas of the brain deputed to the encoding of reward (e.g., insula; Stice et al., 2008). This suggests that cognitive and motivational aspects might have a stronger influence on people suffering from eating disorders than purely perceptual mechanisms.

## FOOD NEOPHOBIA, TASTE, AND OLFACTION

While a number of investigations have been made on the existence of systematic links between individual factors (psychological, demographical, etc.) and levels of food neophobia, others have turned their attention toward the role of sensory functions. For instance starting from the observation that neophobic children mainly refuse fruit and vegetables rather than other food categories (Wardle and Cooke, 2008), Coulthard and Blissett (2009) hypothesized that the rationale behind that could be a higher sensitivity to taste, and to bitter in particular. Using indirect measurements (i.e., parental proxy questionnaires), they highlighted that high taste sensitivity negatively correlated with the amount and variety of consumed fruit and vegetables and with the levels of food neophobia. Adults tested for their sensitivity to phenylthiocarbamide (PTC) or quinine hemisulfate (i.e., bitter substances) revealed though not to differ as a function of their attitude toward novel foods (Frank and van der Klaauw, 1994). Willingness to try unfamiliar foods, rather than having direct effects on sensory perception, influenced the hedonic evaluation of a series of food-related and food-unrelated odors. Almost all odors were judged as being less pleasant and less intense by people reluctant to try new foods supporting the notion of an important role of olfaction in food preferences and eating behavior. Interestingly, neophobic people tend to use smaller sniff magnitudes than non-neophobics, as measured during an odor detection task (Raudenbush et al., 1998), and this has been interpreted as an index of an attempt made by neophobics to avoid any possible bad odor-related experiences (Prescott et al., 2010). This would be consistent with the hypothesis that food neophobia might result from the anticipation of a possible negative outcome produced by tasting the unknown product (Pliner et al., 1993). During uncertain conditions in particular (i.e., when the information available is very scant), neophobics expect to like an unfamiliar food significantly less than neophilics. Compared to this latter group, neophobics appear to feel more uncertain about the identity of the unknown product. They are also less willing to try unfamiliar foods even when a future hypothetical situation is considered (Tuorila et al., 1994; see also Frank and Raudenbush, 1998).

Active exploration of the environment through sniffing is reckoned to be a key factor for odor detection. Frasnelli et al. (2009) described that the ability to localize a pure odorant (that is an odor that does not stimulate the trigeminal system, such as the rose-like odor of phenyl ethyl alcohol) by discriminating the stimulated nostril (right vs. left) varies as a function of the stimulus being actively sniffed or passively perceived (i.e., mechanically delivered into the nostrils). Tourbier and Doty (2007), instead, demonstrated that sniff magnitude correlates with human olfactory abilities as measured by the University of Pennsylvania Smell Identification Test (UPSIT; Doty et al., 1984), with participants having a sense of smell in the normal range showing smaller magnitude sniffs than anosmic participants. In addition interestingly, these authors highlighted that the sniff magnitude ratio is strongly modulated by the hedonic value of the perceived odor (i.e., it decreases when malodor rather than a pleasant odor is used; see also Djordjevic et al., 2008), which suggests a possible important role of expectancy in olfactory behavior that would be mediated by the hedonic dimension of odors.

Odor identification seems to be positively linked to the degree of experience one person has of the olfactory world (Lehrner and Walla, 2002; see also de Wijk and Cain, 1994a; Cain et al., 1995; Lehrner et al., 1999). de Wijk and Cain, (1994b) for instance described that odor identification ability varies according to age, being poor in childhood and improving until adulthood (Cain et al., 1995). This improvement in the odor identification ability is suggested to occur throughout the whole life-span and is dependent on a learning effect induced by a repeated exposure to the different odors. Following this logic, Demattè et al. (2013) recently formulated the hypothesis that the scant exploratory behavior described in food neophobics (Raudenbush et al., 1998) could also affect the ability of finding the right name for an odor. Therefore, a group of adult volunteers were asked to identify a series of common odors and the results revealed that neophobic people were significantly worse in the identification task than non-neophobic participants. A connection thus does seem to exist between the personal attitude toward unknown foods (as measured by the FNS) and the ability to name common odors. This relation would be mediated by the different degree of exposure a person has to different odors during life. Interestingly consistently with this, familiarity appears to have an important role in different aspects of olfactory perception (for a recent review on olfactory expertise, see Royet et al., 2013). An odor never encountered before is usually evaluated as being less pleasant than a more familiar odor (Delplanque et al., 2008), while repeated exposure to an odor appears to lower the threshold for its detection (Dalton et al., 2002).

## THE MEDIATION OF EXPOSURE

The existence of an extremely powerful connection between food neophobia and both the variety in a person’s diet and the repeated exposure to food products has been repeatedly demonstrated (for an earlier review, see Frank and Raudenbush, 1998; see also Pliner et al., 1993; Birch et al., 1998). In adults, diet variety plays a significant impact, as demonstrated by the negative correlation observed between the levels of food neophobia and the levels of both education and socio-economical status (Flight et al., 2003; Meiselman et al., 2010). This effect appears to be directly related to the frequency with which one person experiences different kinds of foods during everyday life (Knaapila et al., 2011). In particular, an increase in the exposure to new food has been proven to reduce general food neophobia levels (Pliner et al., 1993; Birch et al., 1998; Mustonen et al., 2012).

The effects of exposure to different foods on the attitude toward food choices have received special attention in the field of children’s eating behavior (Benton, 2004; Wardle and Cooke, 2008; Raudenbush and Capiola, 2012). A crucial impact of parental behavior on the development of preferences and aversions has been highlighted, both during the weaning phase and later during childhood, and even during a child’s prenatal life (Benton, 2004; Wardle and Cooke, 2008; Beauchamp and Mennella, 2011). Regular pre-exposure to anise flavor through mothers’ diet has shown to be effective in inducing a preference for anise odor in newborn babies (Schaal et al., 2000). Some preferences are innate in nature, for example bitterness aversion or sweetness preference (Mennella et al., 2005; though see Desor et al., 1975), nevertheless prenatal life has been shown to have an impact also on later food preferences, showing the importance of mothers’ diet quality during gestation (Trout and Wetzel-Effinger, 2012).

Food experience in the first period after birth is critical in the learning of food likes and dislikes, as such experiences are considered to drive the following development and expression of human behavior toward food (Beauchamp and Mennella, 2009). Sullivan et al. (1991) for instance have described that 1 day after birth, newborns can already learn to associate pairs of simultaneous olfactory and tactile stimuli, showing a conditioned response for the single conditioned odor experienced before. Later on during weaning, the repeated exposure to a food dramatically influences its acceptance (Nicklaus, 2011). This seems to be true if the food is actually tasted, as mere visual exposure is not sufficient to shape that preference. Other studies have highlighted the importance of parental eating style, that can influence children’s food preferences by determining the ease with which they have access to a sufficiently varied diet (Finistrella et al., 2012) and by means of the powerful mechanism of parental modeling (Benton, 2004; Wardle and Cooke, 2008). In this view, it is not surprising that children’s preferences strongly correlates with those of their mothers (Howard et al., 2012).

## CONCLUSION

Food preferences and aversions are mediated by the chemosensory system, which underlies flavor perception (Frank and van der Klaauw, 1994). The mechanism through which food likes and dislikes are learned and modulated is represented by repeated exposure, but only if it includes actual tasting (Benton, 2004; Wardle and Cooke, 2008). Food neophobia appears to be an extremely complex attitude, its strength fluctuates during life-span and is modulated by a number of different factors (Otis, 1984; Frank and Raudenbush, 1998; Howard et al., 2012). An individual’s diet quality is strongly influenced by the attitude toward food (and novel food in particular) and it has a dramatic impact on her/his health and well-being (Falciglia et al., 2000; Capiola and Raudenbush, 2012). Therefore, an increase in the understanding of the mechanisms underlying food neophobia acquisition and modulation appears to be a critical issue for future investigations.

## Conflict of Interest Statement

The authors declare that the research was conducted in the absence of any commercial or financial relationships that could be construed as a potential conflict of interest.
